# Magnetic graphene oxide–lignin nanobiocomposite: a novel, eco-friendly and stable nanostructure suitable for hyperthermia in cancer therapy

**DOI:** 10.1039/d1ra08640e

**Published:** 2022-01-26

**Authors:** Reza Eivazzadeh-Keihan, Somayeh Asgharnasl, Hooman Aghamirza Moghim Aliabadi, Behnam Tahmasebi, Fateme Radinekiyan, Ali Maleki, Hossein Bahreinizad, Mohammad Mahdavi, Mohammadhossein Shahsavari Alavijeh, Reza Saber, Senentxu Lanceros-Méndez, Ahmed Esmail Shalan

**Affiliations:** Catalysts and Organic Synthesis Research Laboratory, Department of Chemistry, Iran University of Science and Technology Tehran 16846-13114 Iran maleki@iust.ac.ir +98-21-73021584 +98-21-73228313; Protein Chemistry Laboratory, Department of Medical Biotechnology, Biotechnology Research Center, Pasteur Institute of Iran Tehran Iran; Advanced Chemistry Studies Lab, Department of Chemistry, K. N. Toosi University of Technology Tehran Iran; School of Chemistry, College of Science, University of Tehran Tehran Iran; Mechanical Engineering Department, Sahand University of Technology Tabriz Iran; Endocrinology and Metabolism Research Center, Endocrinology and Metabolism Clinical Sciences Institute, Tehran University of Medical Sciences Tehran Iran; Department of Mechanical Engineering, Science and Research Branch, Islamic Azad University Tehran Iran; Research Center for Science and Technology in Medicine, Tehran University of Medical Sciences Tehran Iran; Department of Medical Nanotechnology, School of Advanced Technologies in Medicine, Tehran University of Medical Sciences Tehran Iran; BCMaterials, Basque Center for Materials, Applications and Nanostructures, Martina Casiano, UPV/EHU Science Park Barrio Sarriena s/n Leioa 48940 Spain; Central Metallurgical Research and Development Institute (CMRDI) P. O. Box 87, Helwan Cairo 11421 Egypt a.shalan133@gmail.com ahmed.shalan@bcmaterials.net; IKERBASQUE, Basque Foundation for Science 48009 Bilbao Spain

## Abstract

In this research, a novel magnetic nanobiocomposite was designed and synthesized in a mild condition, and its potential in an alternating magnetic field was evaluated for hyperthermia applications. For this purpose, in the first step, graphene oxide was functionalized with a natural lignin polymer using epichlorohydrin as the cross-linking agent. In the second step, the designed magnetic graphene oxide–lignin nanobiocomposite was fabricated by the *in situ* preparation of magnetic Fe_3_O_4_ nanoparticles in the presence of graphene oxide functionalized with lignin. The resultant magnetic nanobiocomposite possessed certain main properties, including stability and homogeneity in aqueous solutions, making it suitable for hyperthermia applications. The chemical and structural properties of the synthesized magnetic graphene oxide–lignin composite were characterized using FT-IR, EDX, FE-SEM, TEM, TG and VSM analyses. The saturation magnetization value of this magnetic nanocomposite was recorded as 17.2 emu g^−1^. Further, the maximum specific absorption rate was determined to be 121.22 W g^−1^. Given these results, this newly fabricated magnetic nanobiocomposite may achieve considerable performance under the alternating magnetic field in fluid hyperthermia therapy.

## Abstract 2

A novel nanobiocomposite was designed and synthesized under mild conditions to evaluate its potential in hyperthermia therapy.

## Graphical abstract

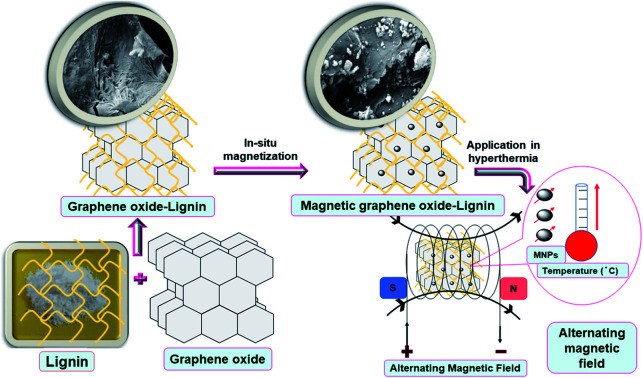

## 1. Introduction

With the discovery and identification of the wide useful properties of graphene, its two-dimensional nanosheet with a hexagonal lattice structure has been considered as one of the most important materials in the scientific world.^1^ Graphene results from very thin layers of graphite. This carbon-based material with a special structure and its derivatives and composites can be applied as supercapacitors, in biosensors, and different other applications.^2,3^ Oxidized derivatives of graphene, such as graphene oxide (GO), can be easily functionalized due to the oxygen-containing functional groups. Besides, the addition of these functional groups to the graphene surface make it an appropriate substrate for fabricating and designing new composites.^4,5^

**Scheme 1:**
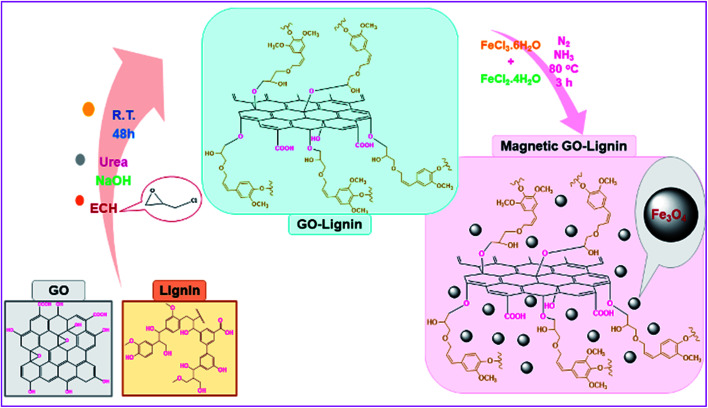
Synthesis process of the magnetic GO–lignin nanobiocomposite.

Nowadays, GO, which is generated from graphite, is one of the most interesting chemical materials among researchers. GO has the high purity of graphene but is much cheaper and much easier to prepare in bulk quantities. Moreover, the existence of oxygen functionalities in the GO structure facilitate other chemicals to grow on it, thereby making GO an important candidate for the fabrication of a wide range of composites and nanocomposites.^6,7^ The modification of GO with different substances has been shown to convert this carbon-based material to useful substrates for various applications.^8,9^ For instance, the functionalization of GO with magnetic nanoparticles changes it to a green and reusable catalyst for multicomponent reactions.^10,11^ On the other hand, GO functionalization is a multifunctional platform to developing adsorption agents for the removal of different kinds of pollutants from aqueous solutions.^12^ In the last decades, drug carriers have gathered great attention in cancer therapy and other branches of medicine towards enhancing the influence of drugs on target cells and reducing the side effect of drug resistance. GO presents high potential for designing versatile structures and drug binding. Therefore, it can be considered an efficient drug delivery system.^13,14^ Moreover, GO composites designed with natural polymers offer biocompatibility and biodegradability. Some of the most important and available polymers that enable efficient conversion on the base substrate are alginate,^15^ cellulose,^16^ chitosan,^17^ starch,^18^ and lignin.^19^ For example, the combination of GO with chitosan generated nanocomposites, which were employed as electrochemical DNA biosensors for the detection of typhoid.^20^ In another example, the combination of GO and natural cellulose lead to nanofiber composite films with strongly anisotropic thermal conductivity.^21^ Besides, supercapacitor composites are another achievement of the reaction between GO and polymers.^22^ Lignin is the main component of lignocellulosic biomass (LCBM); it is a biodegradable, amorphous, irregular three-dimensional, and highly branched phenolic and complex polymer.^23^ After cellulose, lignin, with a three-dimensional network of cross-linked phenylpropanoid subunits, is the most common polymeric structure in softwoods, hardwoods, and grasses.^24^ There are lots of lignin sources on the earth. But, unfortunately, most of them, such as the bark and wood of trees, are unusable. The main but small source of lignin, which is useable and exploitable, is the pulp and paper industry.^25^ Investigations on the chemical structure of lignin have demonstrated that it is made of three kinds of phenylpropanoid monomers; these carbon-based and aromatic monomers undergo radical coupling reactions to form the final complex structure of lignin. The existence of hydroxyl groups on the lignin structure is one of the reasons it is a suitable candidate for the reaction with GO. Despite the existence of the hydroxyl functional groups, lignin is insoluble in water and alcohol, and to induce this natural polymer to react with chemical substrates, it should be dissolved in alkaline solutions.^26^ In recent years, functionalizing natural polymers through green reactions and under mild conditions, such room temperature, green solvents, and short reaction times, as well as utilizing them in medicine as drug delivery agents, for targeted cancer therapy and in other branches has become common.^27^ Many studies have reported the potential of natural polymers is cancer therapy; starch and its nanoparticles are examples.^28,29^ Cancer treatment methods, such as surgery, radiation therapy, chemotherapy, targeted therapy, and hyperthermia, are one of the biggest issues in today's life. However, some of the cancer treatment methods like chemotherapy are as harmful as they are effective due to the side effects. Recently, new treatments have been developed with less side effects.^30^ One of these new remedies is the use of magnetic nanoparticles for hyperthermia treatment.^31–33^ Due to the size-dependent tuneable magnetic feature, large surface area, super-paramagnetic behaviour and other physiochemical properties, applying these highly potent nano-agents as platform materials has been proven promising and ideal in catalysis,^34–36^ water treatment,^37^ and different biomedical fields.^38–41^ The Fe_3_O_4_ magnetic nanoparticles (Fe_3_O_4_ MNPs) present in the structure of magnetic-responsive materials play the main role in the hyperthermia process.^42–44^ In other words, Fe_3_O_4_ MNPs are introduced into the cancer tissue, and by applying an alternating magnetic field (AMF), the death of cancer cells is achieved. In addition to their easy synthesis process, biocompatibility, high penetration depth, and low toxicity, these nanoparticles must have reusability in the tumor region.^45^ After the insertion of MNPs in cancer cells and exerting AMF, the vibration of particles induces Brownian and Néel relaxation, increasing the temperature of the cells.^46^ This increase leads to the killing of the cancer cells. Heating the tumor has two general effects in cancer therapy. The first is, increasing the temperature to 42–46 °C by itself is a way of destroying the tumors. The second is, cell heating causes a better operation of chemotherapy.^47^ The two parameters of Fe_3_O_4_ MNPs essential for the hyperthermia process are concentration and the specific absorption rate (SAR). Moreover, different geometric shapes of nanoparticles effects in hysteresis loss and SAR reduction.^48,49^ In the hyperthermia process, a solution of Fe_3_O_4_ MNPs is prepared and injected into the target cells. Furthermore, the injection time depends on the injection rate. Above all, the prepared sample solution has to be homogenous during the injection process. The hydrophilic structure of the Fe_3_O_4_ MNPs used for inducing hyperthermia can aid in easily meeting this basic requirement.^50^ In this study, natural and non-toxic materials have been used to design a mild and eco-friendly reaction, which generates a hydrophilic and new magnetic nanobiocomposite. In the first synthetic step, GO was synthesized using a modified Hummers' method. Then, it was functionalized with natural lignin (GO–lignin). Following that, the *in situ* magnetization process of GO–lignin was implemented, and the new magnetic GO–lignin nanobiocomposite was prepared (Scheme 1). Given the homogeneity and constant stability in water, as well as its efficient magnetic property, the potency of the magnetic GO–lignin nanobiocomposite was evaluated using an alternating magnetic field (AMF).

## 2. Experimental section

### 2.1. Materials and methods

All the required chemical substances, such as solvents and reagents, were of high purity and bought from Merck, Aldrich and Fluka international companies. The FT-IR spectra were acquired on a Shimadzu IR-470 spectrometer (Japan) by the KBr pellet method. The morphology and structure of the synthesized magnetic nanobiocomposite were characterized by field-emission scanning electron microscopy (FE-SEM) (ZEISS-sigma VP model, Germany) and transmission electron microscopy (TEM) (Zeiss-EM10C-100KV, Germany). The vibrating sample magnetometry (VSM) analysis was performed by using a LBKFB model-magnetic Kashan Kavir (Iran) (5000 Oe). The energy-dispersive X-ray (EDX) analysis was accomplished using a Numerix DXP-X10P (France). The thermogravimetric (TG) analysis and thermal behavior of the magnetic biocomposite were analyzed by a Bahr-STA 504 instrument (Germany) under an argon atmosphere at the heating rate of 10°C min^−1^. Finally, the hyperthermia application of this composite was tested in varied frequencies by the device from NATSYCO, Iran.

### 2.2. Synthesis of GO

A modified Hummers' method with sonication was used to synthesize GO.^51,52^ In this regard, at first 1.00 g of graphite and 0.5 g of NaNO_3_ were added to 23 mL of sulfuric acid (98%), The obtained liquid solution was stirred at 66 °C and then sonicated for 30 min in room temperature. In the next step, 3.00 g of KMnO_4_ was slowly added to the container, which was located in an ice bath, and the suspension was reacted for 1 h in the sonication bath. The temperature of the suspension was increased to 98 °C; then, 50 mL of distilled water was added, and a brown suspension was obtained. After that, 700 mL of warm distilled water was mixed with the brown suspension to dilute the mixture. In the end, 12 mL of H_2_O_2_ was added at once and a colour change from brown to yellow was observed. Finally, 2 mL of HCl and 98 mL of distilled water were added, and after one day, the product was repeatedly washed and dried at 60 °C.

### 2.3. Functionalization of GO using lignin (GO–lignin)

According to a previously reported method,^53^ the coalition of GO and lignin was accomplished in a one-step procedure. In order to link GO to lignin, a solution was prepared according to previous work.^54^ In 100 mL of an aqueous solution containing 7.0 wt% NaOH/12.0 wt% urea/81.0 wt% distilled water, 0.3 g of GO was dispersed for 15 min. Then, 6 mL of epichlorohydrin (ECH), the cross-linking agent, was added to the suspension and stirred for 10 min. Finally, 3.00 g of lignin was added to the mixture, and the solution was kept under stirring for 48 h at room temperature. After the mentioned time, the product was centrifuged (4000 rpm, 5 min), washed with ethanol and dried in a freeze-drier overnight.

### 2.4. Magnetization process of GO–lignin

For the first time, the GO–lignin composite was magnetized using Fe_3_O_4_ nanoparticles *via* an *in situ* procedure in this study. For this, 0.2 g of GO–lignin, 0.97 g FeCl_3_·6H_2_O and 0.43 g FeCl_2_·4H_2_O were added to 200 mL of distilled water in an N_2_ atmosphere, and the mixture was stirred for 30 min at 50 °C. Then, 10 mL of 25% aqueous ammonia was trickled into the solution in an N_2_ atmosphere. The resulting mixture was stirred for 3 h at 80 °C for completing the reaction. After the mentioned time, the obtained new magnetic nanocomposite was separated with an external magnet and washed with distilled water to reach natural pH (pH = 7).

## 3. Results and discussion

As we know, one of the main goals of organic and green chemistry is to design novel nanobiocomposites that are natural-product-based, eco-friendly, easy to fabricate and useful in biomedicine and medical applications. In this research, GO was functionalized with the natural complex polymer named lignin. Since the substance should be magnetic to be responsive to the alternating magnetic field (AMF) and applied in hyperthermia, the fabricated composite was made magnetic by the *in situ* construction of magnetic Fe_3_O_4_ nanoparticles (Scheme 1). The structure and properties of the designed magnetic GO–lignin nanobiocomposite were analysed by a wide range of spectroscopic and analytical techniques. The FT-IR spectra demonstrated the formation of new functional groups and occurrence of the reaction between the reagents; the FE-SEM images revealed the structure and morphology of the functionalized composite and the nanoparticles. The VSM analysis was used to show the magnetic property, and EDX analysis was carried out to determine the elemental combination and the inclusion of new elements in the structure. The TG curve showed the thermal resistance of the composite, and the TEM image illustrated the structure of magnetic GO–lignin.

### 3.1. Characterization of the magnetic GO–lignin nanobiocomposite

#### 3.1.1. FT-IR analysis

**Fig. 1:**
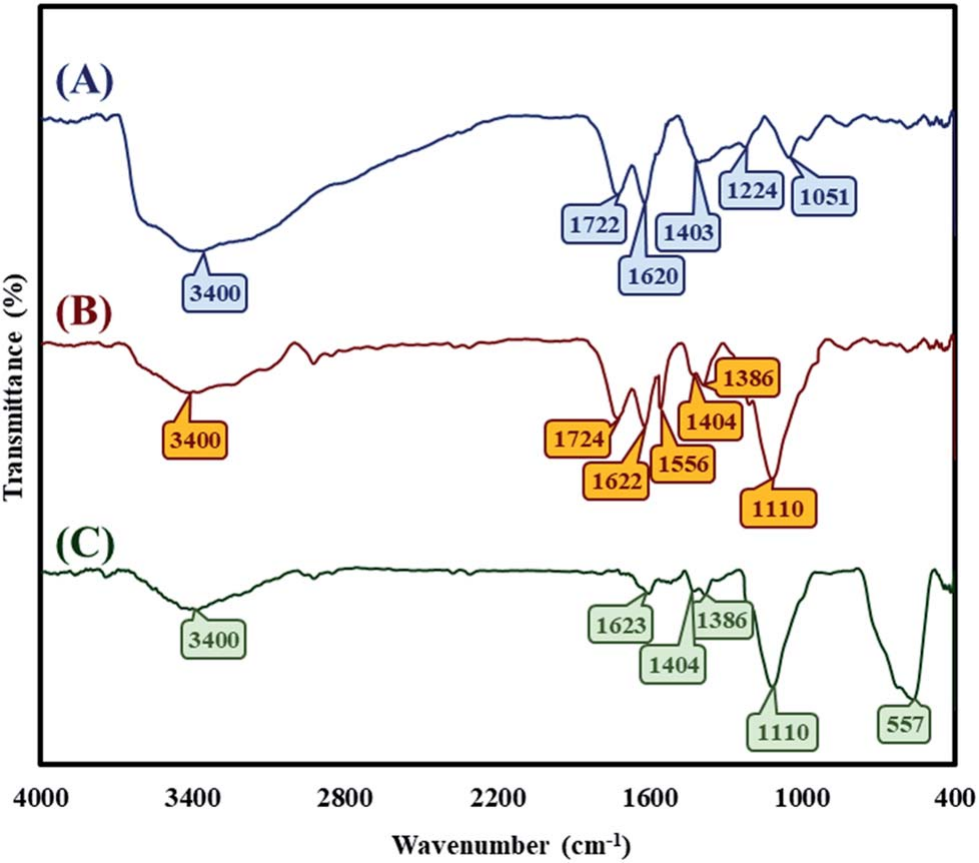
FT-IR spectra of (A) GO, (B) GO–lignin, and (C) the magnetic GO–lignin nanobiocomposite.

The detection of the functional groups of GO, the synthesized GO–lignin composite and also the newly fabricated magnetic GO–lignin nanobiocomposite was achieved by the FT-IR technique. As shown in Fig. 1A, there was a broad and strong band at 3200–3600 (3400) cm^−1^ related to the stretching vibration of the O–H of GO; the other bands at 1722, 1620, 1224, 1051, and 1403 cm^−1^ belonged to the stretching vibration of the carbonyl and carboxyl (C=O, C–OH and C–O) functional groups of GO,^55^ as well as the O–H deformation vibration mode.^56^ In Fig. 1B, the strong and wider absorption band at 1022–1198 cm^−1^ shows the formation of GO–lignin and demonstrates the presence of ether functional groups. The aromatic skeletal vibration mode of lignin can be observed at 1556 cm^−1^.^57^ Moreover, the small band at 1386 cm^−1^ could be attributed to the vibration mode of the syringyl and guaiacyl rings of lignin.^58^ In addition to this, the peak of the C–O functional groups of GO overlapped with the newly synthesized functional groups.^59^ Furthermore, the presence of a strong absorbance band at 557 cm^−1^ in Fig. 1C is because of the addition of the magnetic Fe_3_O_4_ nanoparticles to the composite.

#### 3.1.2. EDX analysis

**Fig. 2:**
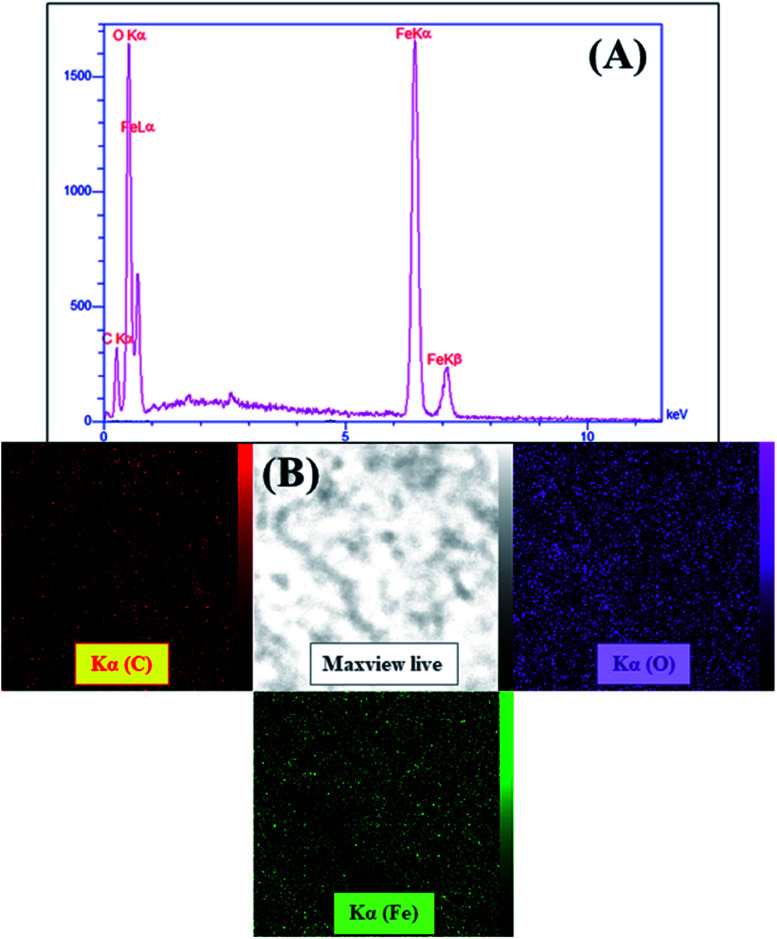
(A) The EDX spectrum and (B) elemental mapping images of the magnetic GO–lignin nanobiocomposite.

Given the capacity of EDX analysis in detecting the constitutive elements in a wide range of organic and inorganic materials, the elemental composition of magnetic GO–lignin was well-determined by its EDX spectrum (Fig. 2A). Further, the elemental mapping of this magnetic nanobiocomposite is shown in Fig. 2B to reveal the distribution of the elements mentioned in the EDX spectrum. The presence of the carbon and oxygen peaks referred to the basic structures of GO and lignin and the oxygen atoms in Fe_3_O_4_ MNPs. The two peaks of Fe asserted the fabrication of the magnetic Fe_3_O_4_ nanoparticles and magnetization of the GO–lignin substrate.

#### 3.1.3. FE-SEM imaging

**Fig. 3:**
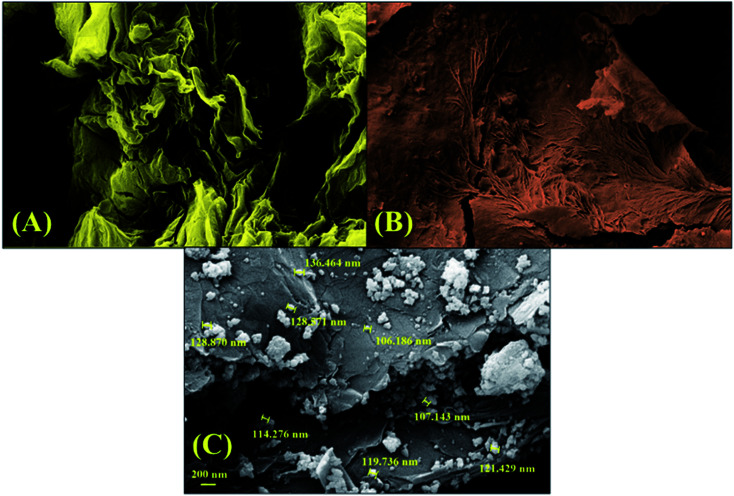
FE-SEM images of (A) GO, (B) GO–lignin, and (C) the magnetic GO–lignin nanobiocomposite.

FE-SEM imaging was used to evaluate the morphology and construction of GO, GO–lignin and the magnetic GO–lignin nanobiocomposite. Moreover, FE-SEM imaging revealed the distribution of the magnetic Fe_3_O_4_ nanoparticles in the fabricated GO–lignin nanobiocomposite. As illustrated in Fig. 3A, the GO nano-sheets were laminated, and a uniform formation on the surface could also be seen. The FE-SEM image of the designed GO–lignin is presented in Fig. 3B. As seen, some wrinkles with different patterns had appeared, and these could be attributed to the reaction between lignin and GO and the formation of the GO–lignin substrate. Besides, given the observations from Fig. 3C, the formation of the GO–lignin nanobiocomposite and the *in situ* preparation of Fe_3_O_4_ nanoparticles were accompanied by obvious changes in morphology. The synthesized Fe_3_O_4_ nanoparticles were uniformly distributed on the surface of GO–lignin. In addition, the average size of these nanoparticles was estimated to be between 106 and 136 nm.

#### 3.1.4. TEM imaging

**Fig. 4:**
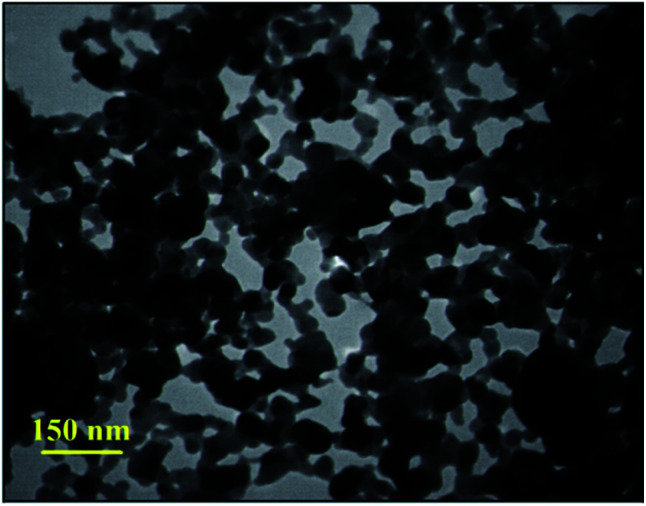
TEM image of the magnetic GO–lignin nanobiocomposite.

The TEM imaging results exhibited high correspondence with the other analyses. As seen in Fig. 4, the observed dark black points were related to the Fe_3_O_4_ MNPs synthesized inside the GO–lignin substrate *via* an *in situ* preparation process. In addition, these almost uniform nanoparticles were well-distributed in the GO–lignin structure without any agglomeration.

#### 3.1.5. Thermogravimetric analysis

**Fig. 5:**
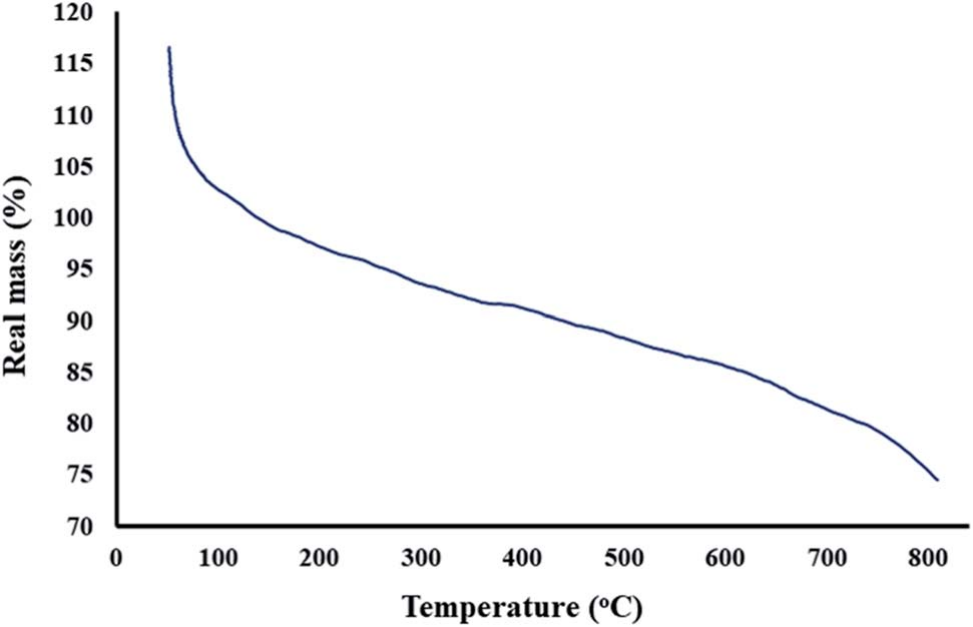
TG curve of the magnetic GO–lignin nanobiocomposite.

Thermal analysis was performed for the investigation of the thermal stability of the synthesized magnetic GO–lignin nanobiocomposite. To assess the thermal endurance of this new nanobiocomposite, the analysis was conducted at the temperature range of 50 °C to 850 °C and the rate of 10°C min^−1^ under an argon atmosphere. As depicted in Fig. 5, the TG curve of the magnetic GO–lignin nanobiocomposite was analogous to the lignin TG curve. For example, in the lignin TG curve, the first step of decrease occurs at about 20–150 °C because of the desorption of physically bound water from the product surface. Besides, the third weight loss in the lignin TG curve occurred in the temperature range between 600–1000 °C similar to the nanobiocomposite TG curve.^60^ However, there was a noticeable difference due to the combination of lignin with graphene oxide and the magnetic nanoparticles. Previous studies have shown that the second weight loss due to the thermal decomposition of in lignin occurs at about 150–600 °C, but the fabricated nanobiocomposite presented a curve with less weight loss in the mentioned range, and hence it can be concluded that the reaction of the oxygen-containing groups between lignin and graphene oxide can be a reason for this observation. Consequently, it can be deduced that the thermal stability of lignin increases in the magnetic GO–lignin nanobiocomposite.

#### 3.1.6. VSM analysis

**Fig. 6:**
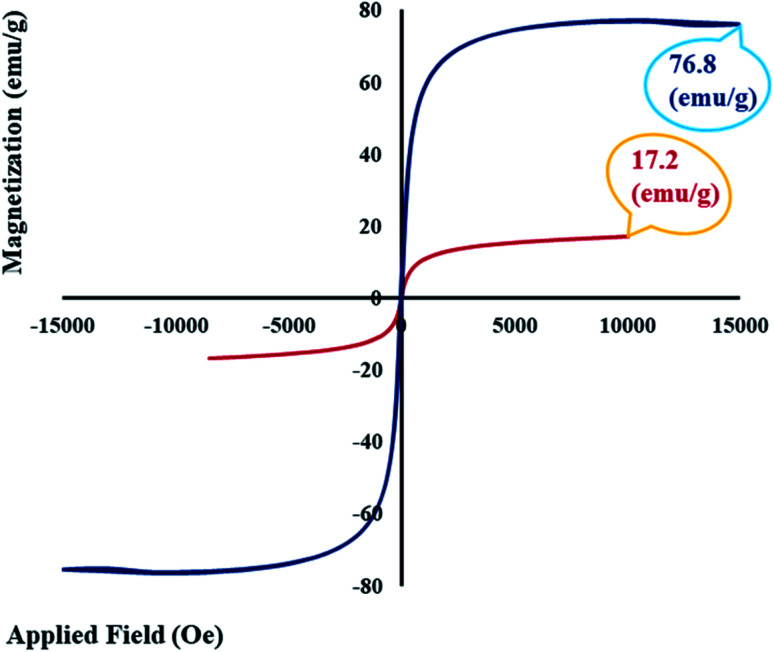
Hysteresis loop curves of bare Fe_3_O_4_ MNPs and the magnetic GO–lignin nanobiocomposite.

As reported in previous studies, the saturation magnetization value (*M*_s_) and magnetic behaviour of magnetic core–shell substances can be influenced by the structure of the nanoparticles, such as core size, shell thickness, and the interparticle and intraparticle interactions.^61^ In Fig. 6, the hysteresis loop curve of magnetic GO–lignin is shown, and the value of saturation magnetization for the designed nanocomposite was about 17 emu g^−1^. Compared with the *M*_s_ value of the bare magnetic Fe_3_O_4_ nanoparticles (76 emu g^−1^), an obvious decrease could be observed. This reduction can be attributed to the placement of the magnetic Fe_3_O_4_ nanoparticles inside the structure of functionalized GO (GO–lignin).

### 3.2. Application of the magnetic GO–lignin nanobiocomposite in the hyperthermia procedure

#### 3.2.1. Heating capacity of the magnetic GO–lignin nanobiocomposite under the AMF

**Fig. 7:**
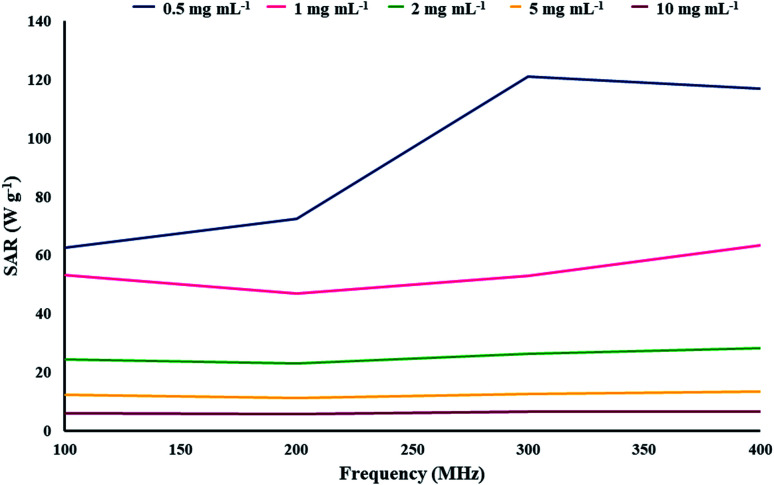
Relation between the SAR and different concentrations of the synthetic magnetic GO–lignin nanocomposite at different AMR frequencies.

When certain nanoparticles get exposed to an alternating magnetic field (AMF), they generate heat through hysteresis and relaxation losses. This effect is dependent on the structure and composition of the magnetic nanoparticles (MNPs), as well as their concentration in the sample and the properties of the magnetic field. Due to the small size and high surface-to-body ratio, these nanoparticles have become a popular choice for inducing hyperthermia in biomedical applications. We can take advantage of this thermal energy by implanting MNPs deep in the tumor tissues. There they can be used to raise the tumor temperature to a specific value depending on the application. Below 41 °C, hyperthermia (mild hyperthermia) is used to improve tumor blood flow, which makes it more sensitive to radio or chemotherapy. Hyperthermia at 42–46 °C can damage the cancer cells while causing minimal damage to healthy cells. However, raising the tissue temperature above these limits can cause necrosis and should be avoided. As the temperature range of hyperthermia for each application is very different, it is essential to control the temperature increase caused by MNPs. In order to control the treatment temperature, we need to know the heating power of the MNPs and understand its variance due to other factors. In this study, the specific absorption rate (SAR) of the MNPs was calculated by eqn (1):1
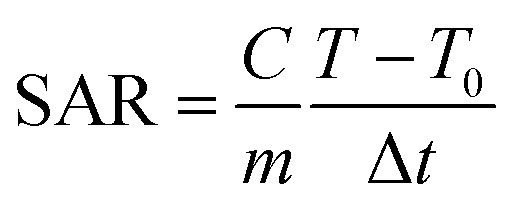
where *C* is the specific heating capacity of the sample, *m* is the concentration of MNPs in the sample, *T* is the temperature of the sample at any given time, *T*_0_ is the initial temperature of the MNPs, and Δ*t* is the time duration. Each sample was kept at a temperature of 25 °C before the test. In this study, the obtained SAR values ranged from 121.22 W g^−1^ (at 0.5 mg mL^−1^ concentration and 300 MHz AMF frequency) to less than 5.68 W g^−1^ (at 10 mg mL^−1^ concentration and 200 MHz AMF frequency). This wide range of thermal power associated with one type of nanoparticle shows the importance of these parameters in the thermal response of the MNPs (Fig. 7). As illustrated in Fig. 7, the MNP concentration has a much higher impact on their thermal performance compared with the AMF frequency because in no case, the sample with a higher concentration could surpass the SAR of the sample with a lower concentration. A decline in the performance of the MNPs with an increase in their concentration in the sample was expected as the dipole–dipole interactions between the nanoparticles can hinder their performance. Furthermore, for every sample, except in the case of 0.5 mg mL^−1^, the maximum SAR was recorded at the maximum AMF frequency. In all these cases, we could see a drop in SAR from 100 to 200 MHz, beyond which it increased until maxing out at 400 MHz. In the 0.5 mg mL^−1^ sample, we could see a steady increase in SAR with an increase in the AMF frequency except from 300 to 400 MHz, where it slightly dropped. These results suggest that magnetic graphene oxide–lignin composite generates more energy under an AMF in the 100–400 MHz frequency range.

#### 3.2.2. The assessment of SAR variation over time with different concentrations of the synthetic magnetic GO–lignin nanobiocomposite

**Fig. 8:**
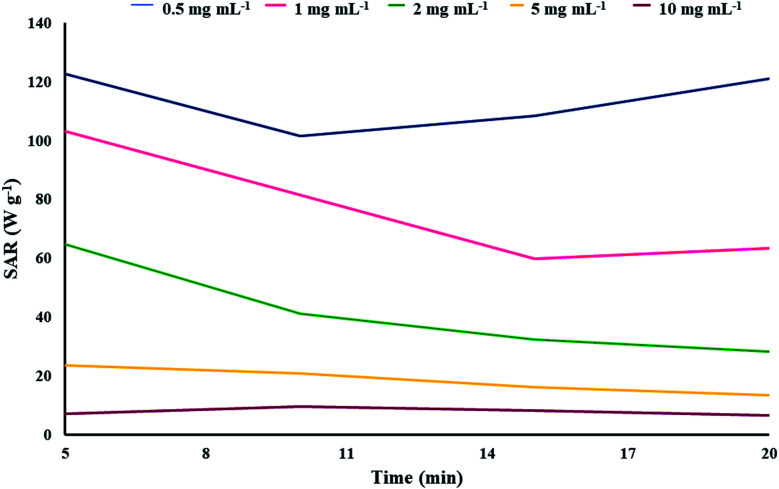
Variation of the maximum SAR of each magnetic sample with time.

The plot of the maximum SAR of each sample *versus* time is shown in Fig. 8. In order to assess the variation of SAR with time, the AMF frequency of 400 MHz was applied for all samples except the 0.5 mg mL^−1^ sample, for which 300 MHz was chosen as this was its maximum SAR. The temperature was recorded *via* a thermocouple at 5 min intervals. For all samples except 10 mg mL^−1^, the maximum SAR was obtained at the 5 min point. While both 2 and 5 mg mL^−1^ samples had a steady decline in SAR, the 0.5 and 10 mg mL^−1^ samples behaved differently. The lower concentration sample had an initial decline in SAR, which then rose until it almost matched its initial value. The higher concentration sample showed a SAR increase from the 5 to 10 min points, but it decreased from then on to eventually a lower number than its initial SAR. Overall, magnetic GO–lignin displayed a wide range of heating power, which makes it a very versatile tool for MNP-mediated hyperthermia. SARs above 100 W g^−1^ can be used to directly damage the cancer cells, while lower SARs could be useful in other applications, such as mild hyperthermia.

#### 3.2.3. Comparative evaluation of the magnetic GO–lignin nanobiocomposite with former reports in the hyperthermia process

In order to investigate the efficiency and heating capacity of the magnetic graphene oxide–lignin nanocomposite, an alternating magnetic field (AMF) was used. The specific absorption rate (SAR) was the main parameter characterized by using five different concentrations. The highest value of SAR (121.22 W g^−1^) was obtained at the lowest concentration (0.5 mg mL^−1^). Moreover, the relationship between time and SAR was studied, and the result showed that at the lowest concentration and the least time presented the highest SAR. In comparison with the previous materials summarized in Table 1, the newly synthesized magnetic GO–lignin nanocomposite appears to have a higher potential in hyperthermia applications. It can also be concluded that the new graphene oxide-based nanocomposite is more important than the other graphene oxide materials. This property is due to its colloidal stability and considerable SAR values at a very low concentration.^62–65^

**Table 1:** The comparative evaluation of the magnetic GO–lignin nanobiocomposite with other nanomaterials reported for use in the hyperthermia process

Entry	MNPs	Nanocomposite	Optimum concentration of nanocomposite (mg mL^−1^)	SAR (W g^−1^)	Magnetic saturation (emu g^−1^)	Ref.
1	Fe_3_O_4_	Magnetic Fe_3_O_4_	15	18.5	58.8	45
2	Fe_3_O_4_	Poly (acrylic acid) coated Fe_3_O_4_	15	46.64	72.38	62
3	Fe_3_O_4_	Oleate coated Fe_3_O_4_	15	51.90	72.38	62
4	Fe_3_O_4_	Brick-like Ag@Fe_3_O_4_	1	100	37	63
5	Fe_3_O_4_	Fe_3_O_4_–chitosan	2	118.85	49.96	64
6	Fe_3_O_4_	Graphene oxide–Fe_3_O_4_	1	98	78	65
7	Fe_3_O_4_	Magnetic GO–lignin	0.5	121.22	17.2	This work

## 4. Conclusions

In this study, the three-dimensional complex polymer lignin was coupled with graphene oxide. Epichlorohydrin (ECH), a colourless liquid, was used as the cross-linking agent to bind GO and lignin and thereby design a new nanocomposite for use in the hyperthermia process. Lignin is the main compound that makes up plants and hence is natural, non-toxic and biodegradable and has the potential for use in medicine and drug-related applications. The fabricated composite of GO and lignin was magnetized, for the first time, in this study. Being magnetic, stable and homogenous in aqueous solutions would make it a suitable material for hyperthermia. Some analyses, including FT-IR, EDX, FE-SEM, TEM, TG, and VSM, demonstrated that the desired nanocomposite was formed. The nanocomposite performance and data from the alternating magnetic field (AMF) showed that the resultant substrate has the potential to be used in hyperthermia. Considering the high stability and good homogeneity of the magnetic GO–lignin nanobiocomposite in the aqueous phase, as well as the high specific absorption rate (121.22 W g^−1^) at the lowest concentration of the magnetic GO–lignin nanobiocomposite (0.5 mg mL^−1^) and the presence of the natural lignin polymer in the structure, the designed magnetic GO–lignin nanobiocomposite seems prospective. However, it must be evaluated more, and *in vivo* studies are needed to determine its applicability in cancer hyperthermia therapy. Furthermore, combining with therapeutic drugs, bioceramics, and other biomaterials may further develop the properties of our designed magnetic GO–lignin nanobiocomposite for application in drug delivery, tissue engineering, and other regenerative medicine strategies.

## Author contributions

R. E. K. and S. A. helped in preparing the material, characterization, and writing the manuscript. H. A. M. A., B. T., F. R. and H. B. helped in characterization of the obtained materials as well as the discussion of the obtained results. M. M., M. S. A. and R. S. participated in plotting the gained data in addition to interpreting these data. Furthermore, A. M., S. L.-M. and A. E. S. designed the research, contributed to supervising the work, discussed the results, and wrote the manuscript. All the authors participated in writing, editing, and revising the manuscript.

## Conflicts of interest

The authors declare no conflict of interest.

## Supplementary Material
